# Supplementary figures and images for: Selective enhancement of low-gamma activity by tACS improves phonemic processing and reading accuracy in dyslexia
Source: PLoS Biol. 2020 Sep 8;18(9):e3000833. doi: 10.1371/journal.pbio.3000833 (PMC7478834; doi:10.1371/journal.pbio.3000833)

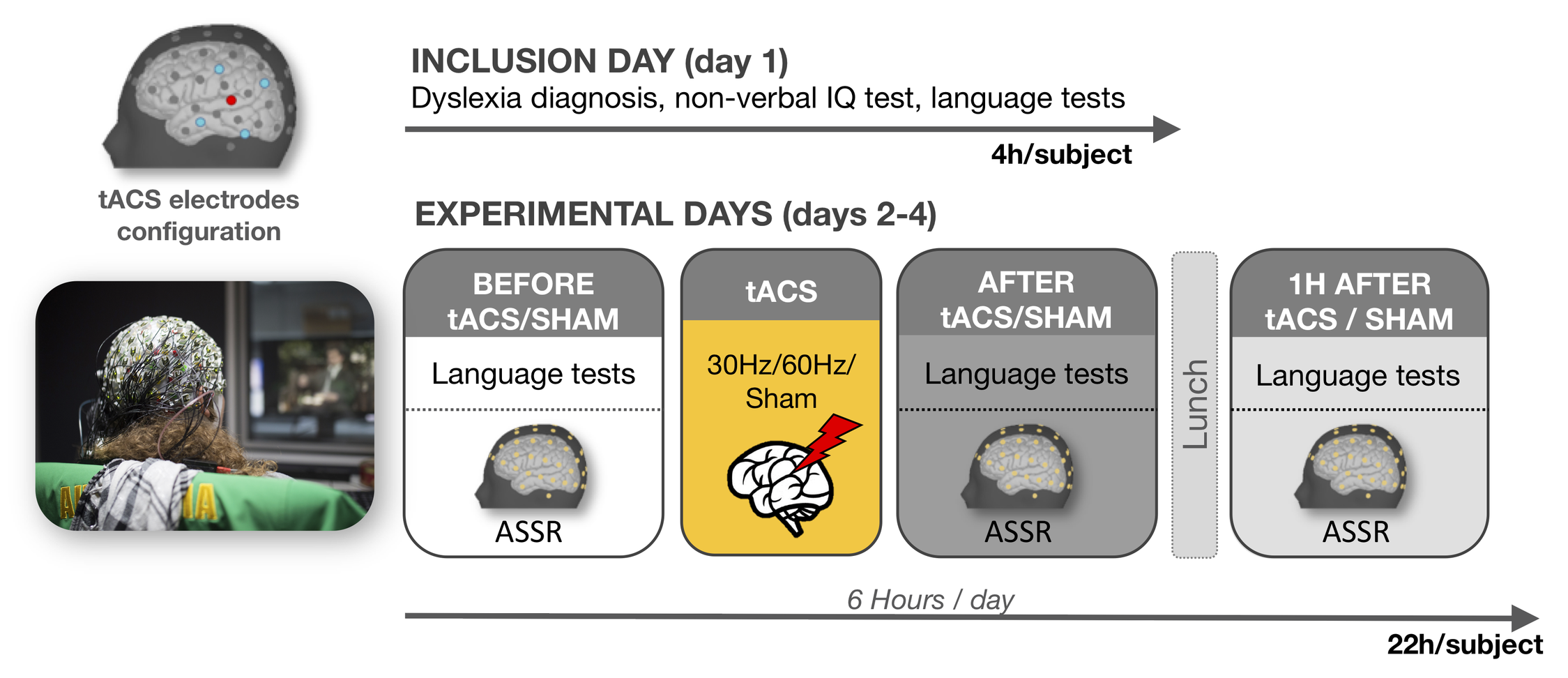

Supplement: S1 Fig — Each participant (15 with dyslexia, 15 without dyslexia) undertook 4 days of testing. During the inclusion day (day 1), the severity of dyslexia was assessed with the ECLA16+ test together with evaluating baseline performance on 3 language tests. These included the pseudoword repetition test, spoonerism test, and text reading, probing, among other things, errors at the phonemic and syllabic level. This allowed us to evaluate phonological awareness, verbal short-term memory, and reading accuracy, as well as the ability to convert lexical orthography to phonology. During each of the following experimental days (days 2–4), one of the 3 tACS stimulation conditions (sham, 30 Hz, and 60 Hz) was administered, with the tACS condition order counterbalanced across subjects. The stimulation lasted 20 minutes and was delivered by means of 5 tACS electrodes organized as a 4 × 1 ring and centered over the left auditory cortex. Within each experimental day, performance on the 3 language tests was evaluated at 3 time points: before, immediately after, and 1 hour after tACS stimulation. At each time point and for each tACS condition, EEG was recorded by means of a 64-channel cap. We used ASSRs to AM pure-tone sounds with fixed frequencies (from 28 Hz to 60 Hz) to entrain brain oscillations in a frequency-specific manner. AM, amplitude-modulated; ASSR, auditory steady-state response; ECLA16+, Évaluation de Compétences de Lecture chez l'Adulte de plus de 16 ans; EEG, electroencephalography; tACS, transcranial alternating current stimulation. (TIF) [file pbio.3000833.s001.tif]

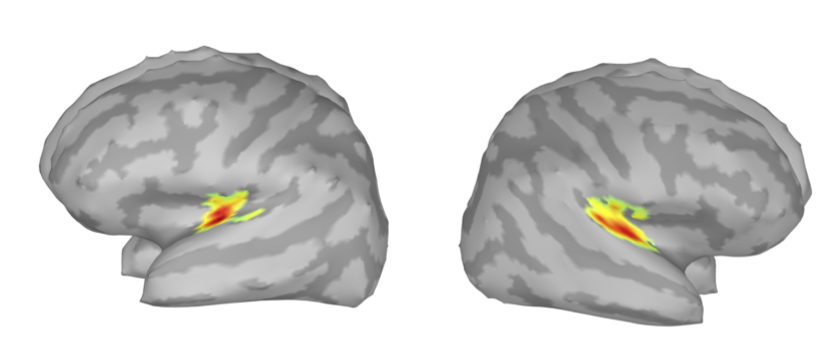

Supplement: S2 Fig — Source localization revealed that the neural generators of the evoked response at short latencies (approximately 60 ms poststimulus onset) are located over the primary auditory cortex bilaterally. (TIF) [file pbio.3000833.s002.tif]

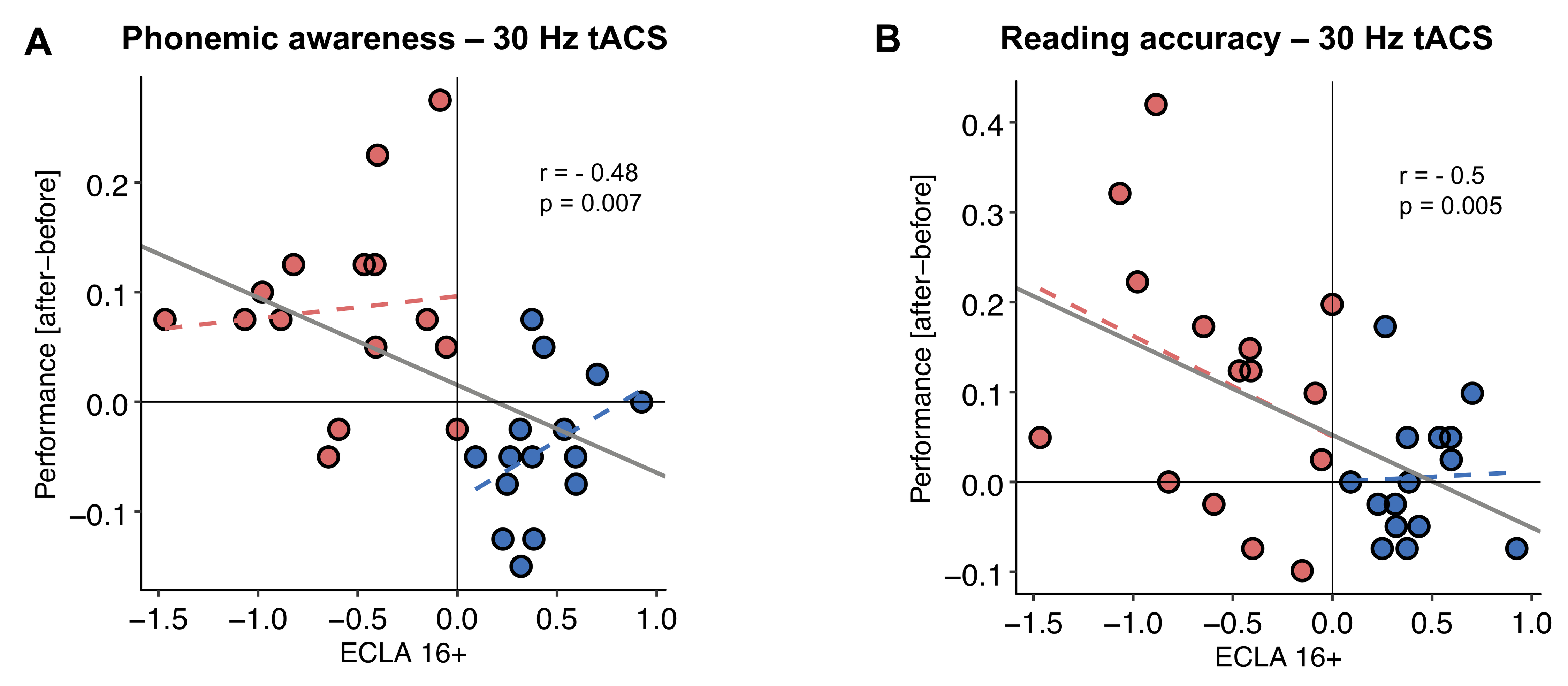

Supplement: S3 Fig — Changes in performance occurring after 30-Hz tACS (difference after/before) for both phonemic awareness (A, r = −0.48, p = 0.007) and reading accuracy (B, r = −0.5, p = 0.005) show a strong negative relationship with language skills (ECLA16+, higher values indicate better reading skills) across groups (solid gray lines). Within-group trend lines are displayed with dashed lines (red: dyslexia group, r = 0.09, p = 0.73 for phonemic awareness, r = −0.32, p = 0.23 for reading accuracy; blue: control group, r = 0.36, p = 0.18 for phonemic awareness, r = 0.034, p = 0.9 for reading accuracy). Numerical data used to generate this figure can be found at https://osf.io/6j49q/. ECLA16+, Évaluation de Compétences de Lecture chez l'Adulte de plus de 16 ans; tACS, transcranial alternating current stimulation. (TIF) [file pbio.3000833.s003.tif]

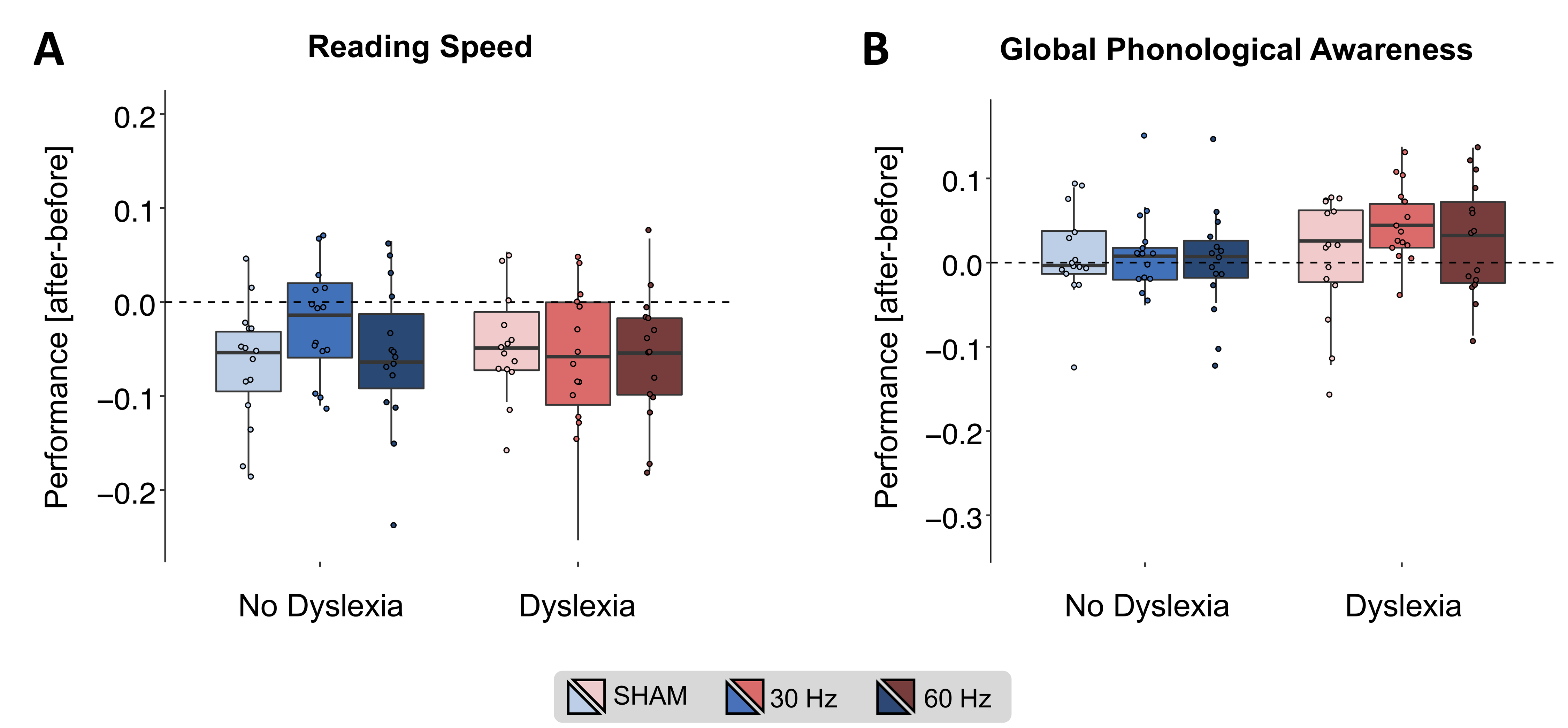

Supplement: S4 Fig — Changes in performance immediately after tACS for each condition (sham, 30 Hz, 60 Hz) in the no-dyslexia (shades of blue) and dyslexia (shades of red) groups are calculated as the difference with respect to the performance measured before the tACS. For reading speed and global phonological awareness, we performed a repeated-measures ANOVA with group (dyslexia, no dyslexia) as a between-subjects factor and stimulation condition (sham, 30 Hz, 60 Hz) as a within-subject factor and found no statistically significant changes. Reading speed: group, F1,28 = 0.02, p > 0.05, η2p = 0.001; tACS condition, F2,56 = 0.38, p > 0.05, η2p = 0.013; interaction group × tACS condition, F2,56 = 2.38, p > 0.05, η2p = 0.07. Global phonological awareness: group, F1,28 = 1.2, p > 0.05, η2p = 0.05; tACS condition, F2,56 = 1.57, p > 0.05, η2p = 0.05; interaction group × tACS condition, F2,56 = 1.09, p > 0.05, η2p = 0.03. Numerical data used to generate this figure can be found at https://osf.io/6j49q/. tACS, transcranial alternating current stimulation. (TIF) [file pbio.3000833.s004.tif]

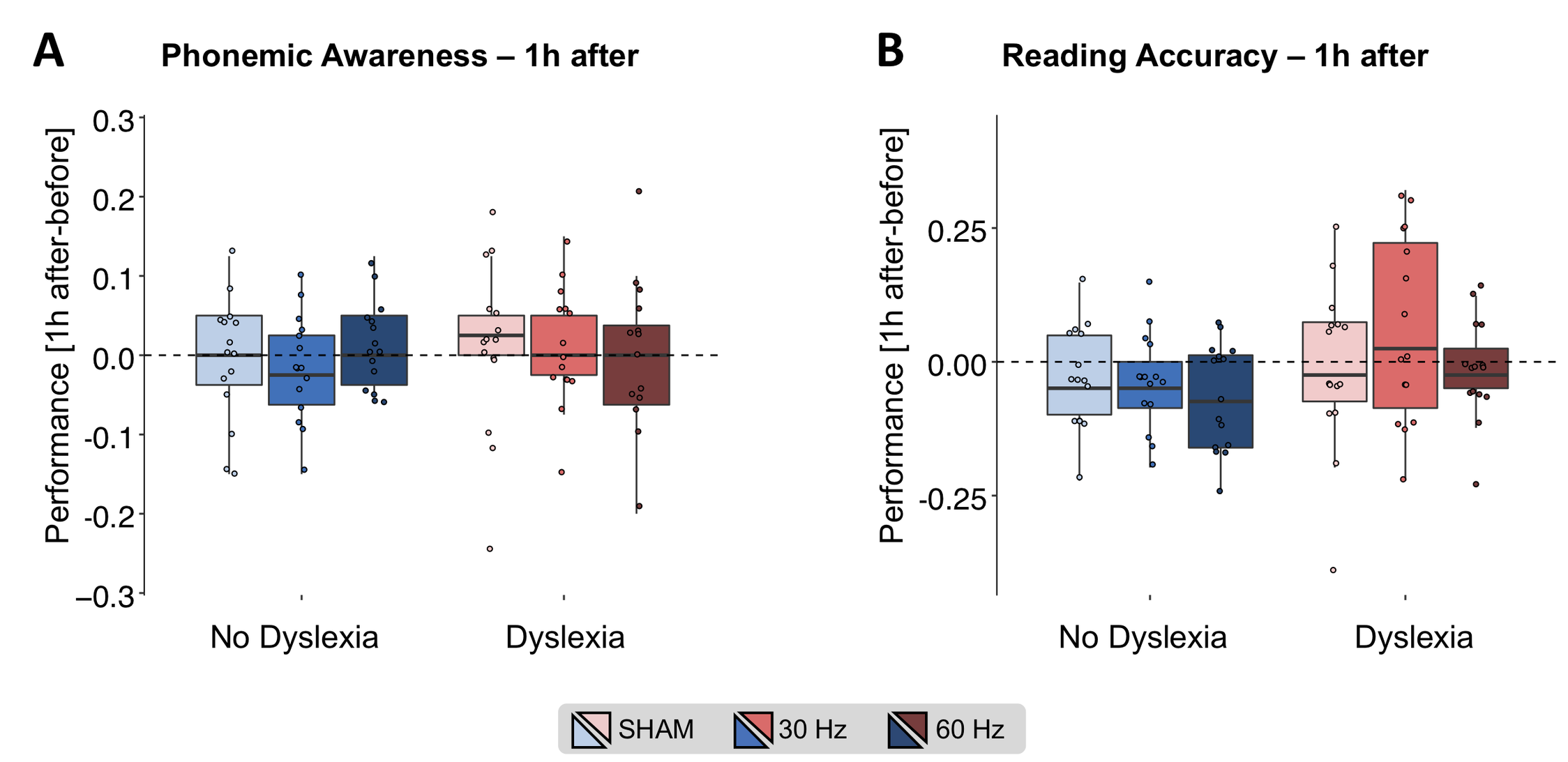

Supplement: S5 Fig — Changes in performance occurring 1 hour after tACS on phonemic awareness (A) and reading accuracy (B) for each tACS condition (sham, 30 Hz, 60 Hz) in the no-dyslexia (shades of blue) and dyslexia (shades of red). Each parameter is calculated as the difference with respect to the performance measured before tACS. For each of the two metrics, we performed a repeated-measures ANOVA with group (dyslexia, no dyslexia) as a between-subjects factor and stimulation condition (sham, 30 Hz, 60 Hz) as a within-subject factor and found no statistically significant changes. Phonemic awareness: group, F1,28 = 0.02, p > 0.05, η2p = 0.001; tACS condition, F2,56 = 0.08, p > 0.05, η2p = 0.003; interaction group × tACS condition, F2,56 = 1.63, p > 0.05, η2p = 0.05. Reading accuracy: group, F1,28 = 2.93, p > 0.05, η2p = 0.11; tACS condition, F2,56 = 2.07, p > 0.05, η2p = 0.09; interaction group × tACS condition, F2,56 = 1.23, p > 0.05, η2p = 0.04. Numerical data used to generate this figure can be found at https://osf.io/6j49q/. tACS, transcranial alternating current stimulation. (TIF) [file pbio.3000833.s005.tif]

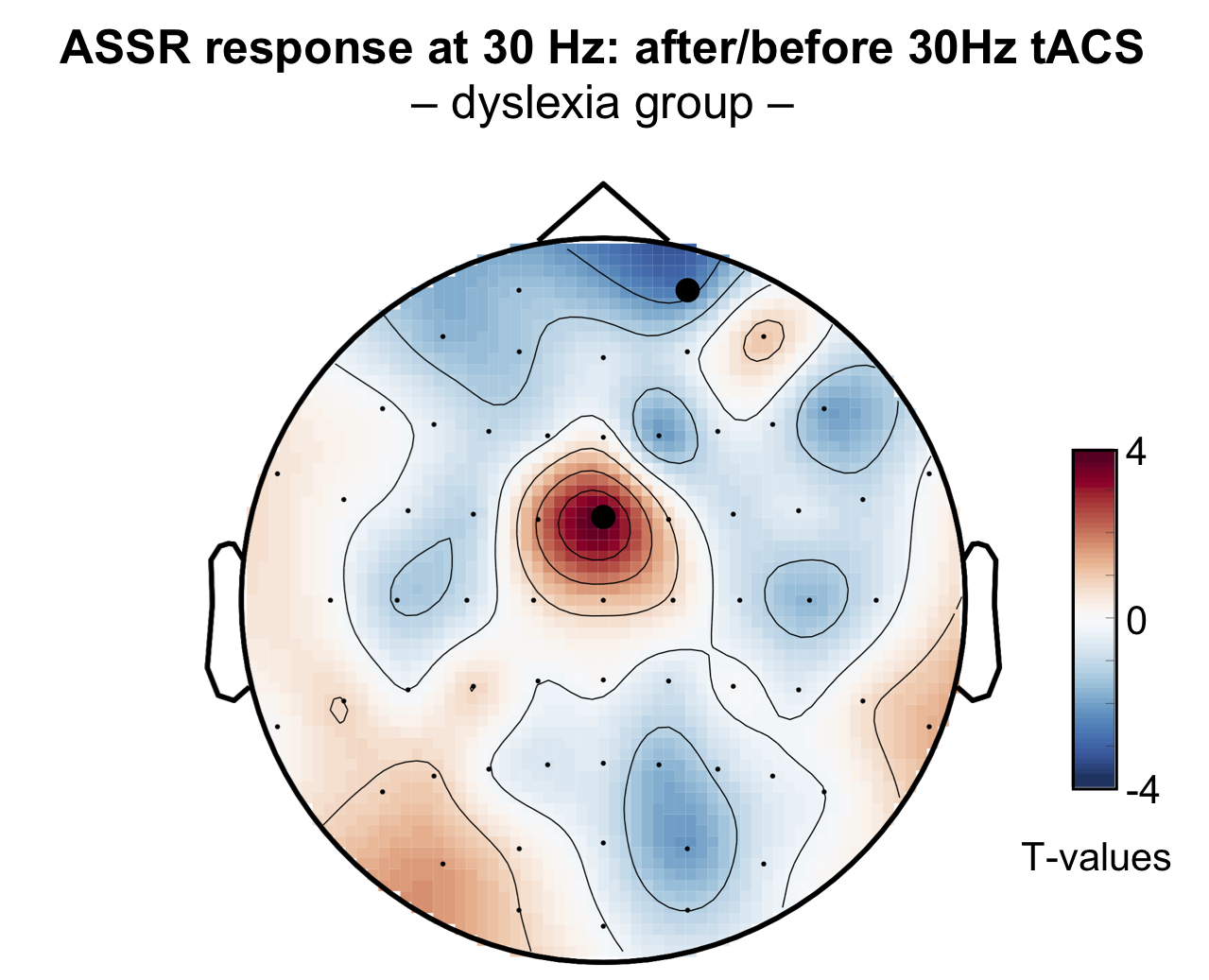

Supplement: S6 Fig — The topography displays t values for each of the 64 channels obtained through 2-tailed paired-samples t test. Positive values indicate a stronger response after the 30-Hz tACS; black dots highlight electrodes with p < 0.05. Numerical data used to generate this figure can be found at https://osf.io/6j49q/. tACS, transcranial alternating current stimulation. (TIF) [file pbio.3000833.s006.tif]

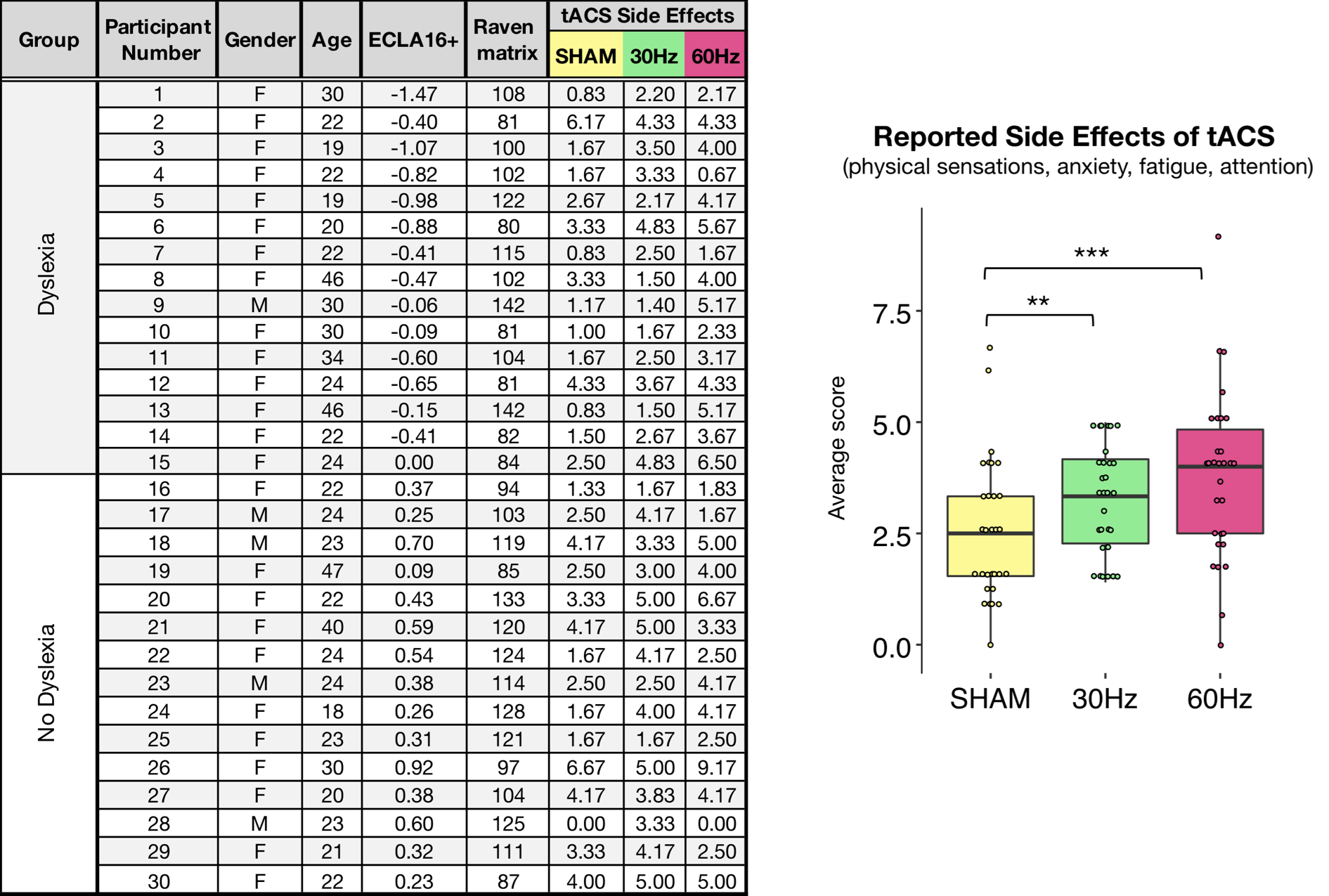

Supplement: S7 Fig — Individual demographic information, language skills measured during the inclusion day (ECLA16+), nonverbal intelligence (Raven matrix), and reported side effects of tACS for each stimulation condition (sham/30 Hz/60 Hz). This included physical sensations—such as pain, warming, tingling—and anxiety, fatigue, and attention. The reported side effects were stronger for the active conditions as compared with the sham stimulation. There was no difference between the dyslexia and no-dyslexia groups. Numerical data used to generate this figure can be found at https://osf.io/6j49q/. Significance is denoted with ** for p < 0.01, *** for p < 0.001. ECLA16+, Évaluation de Compétences de Lecture chez l'Adulte de plus de 16 ans; tACS, transcranial alternating current stimulation. (TIF) [file pbio.3000833.s007.tif]

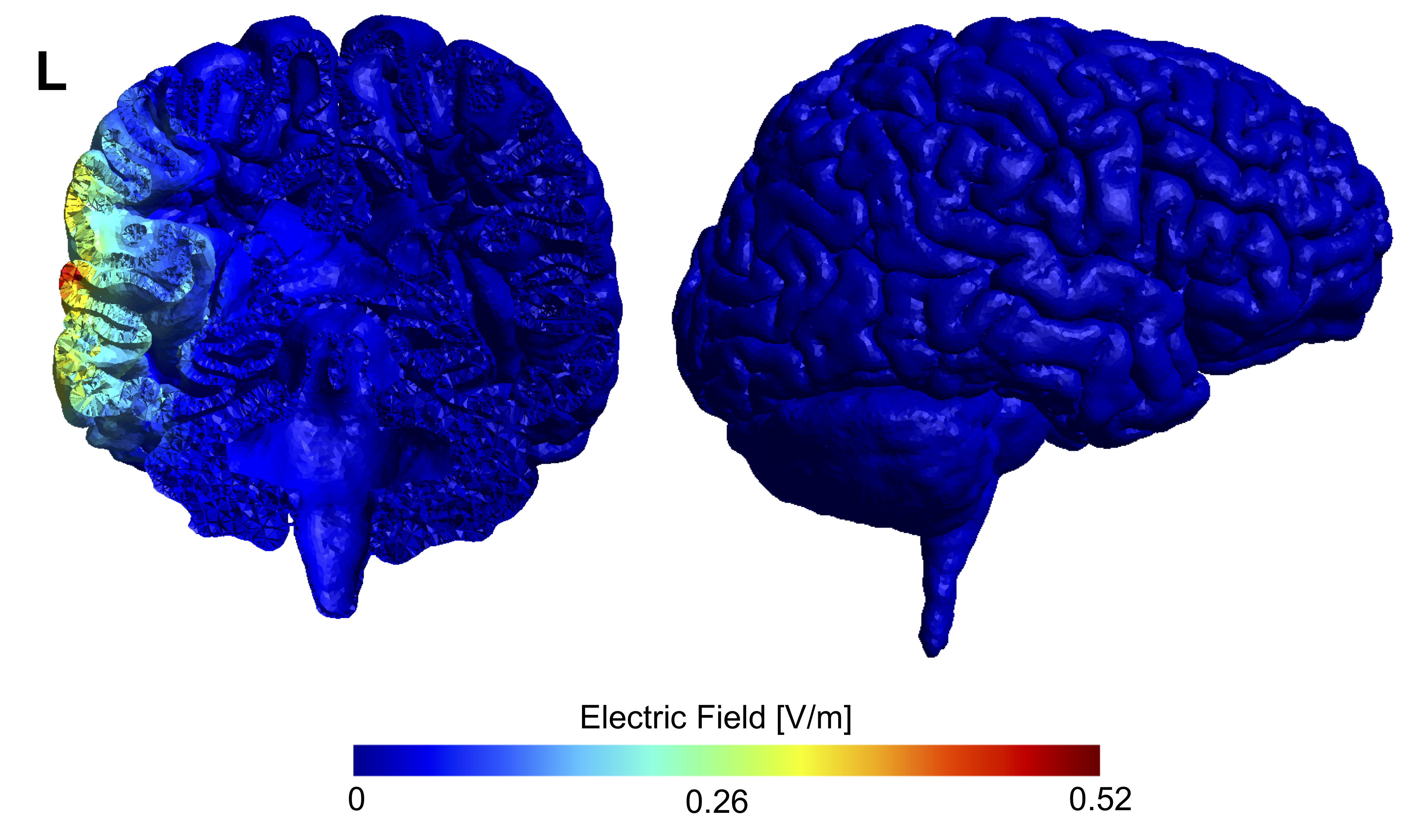

Supplement: S8 Fig — Results show null electric field in the right hemisphere, contralateral to the stimulation site. The coronal view at the peak strength shows focal activity over left auditory regions. tACS, transcranial alternating current stimulation. (TIF) [file pbio.3000833.s008.tif]

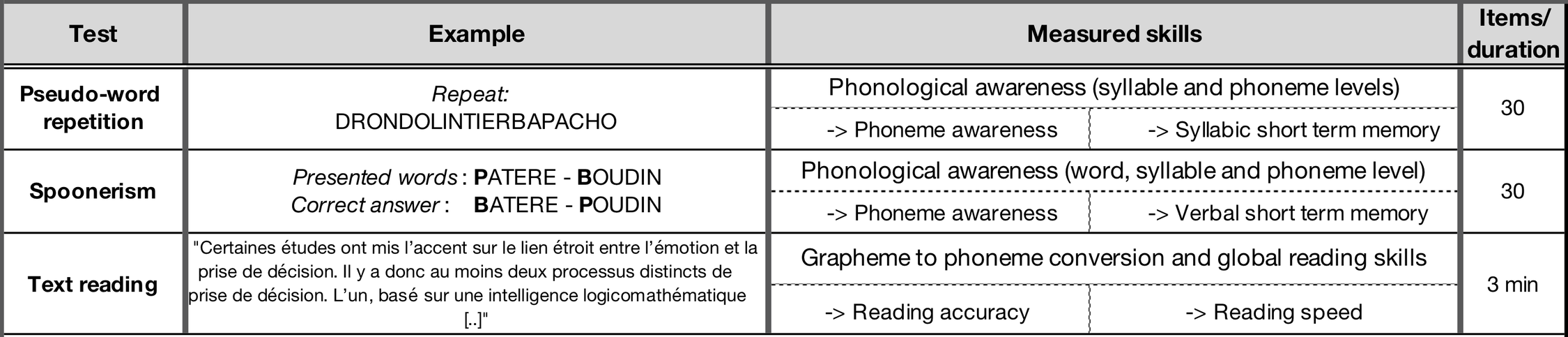

Supplement: S1 Table — Three language tests were designed by a certified linguist to probe phonological processing, short-term memory, and reading skills (speed and accuracy). The pseudoword repetition test consisted of repeating nonlexical words that contain existing syllables in French. The spoonerism test consisted in transposing the first phoneme of 2 words, chosen to have similar phonological and syntactic features and with the same lexical frequency across the 3 subtests, one for each time measurement. Text reading consisted of reading a scientific text for 3 minutes. (TIF) [file pbio.3000833.s009.tif]

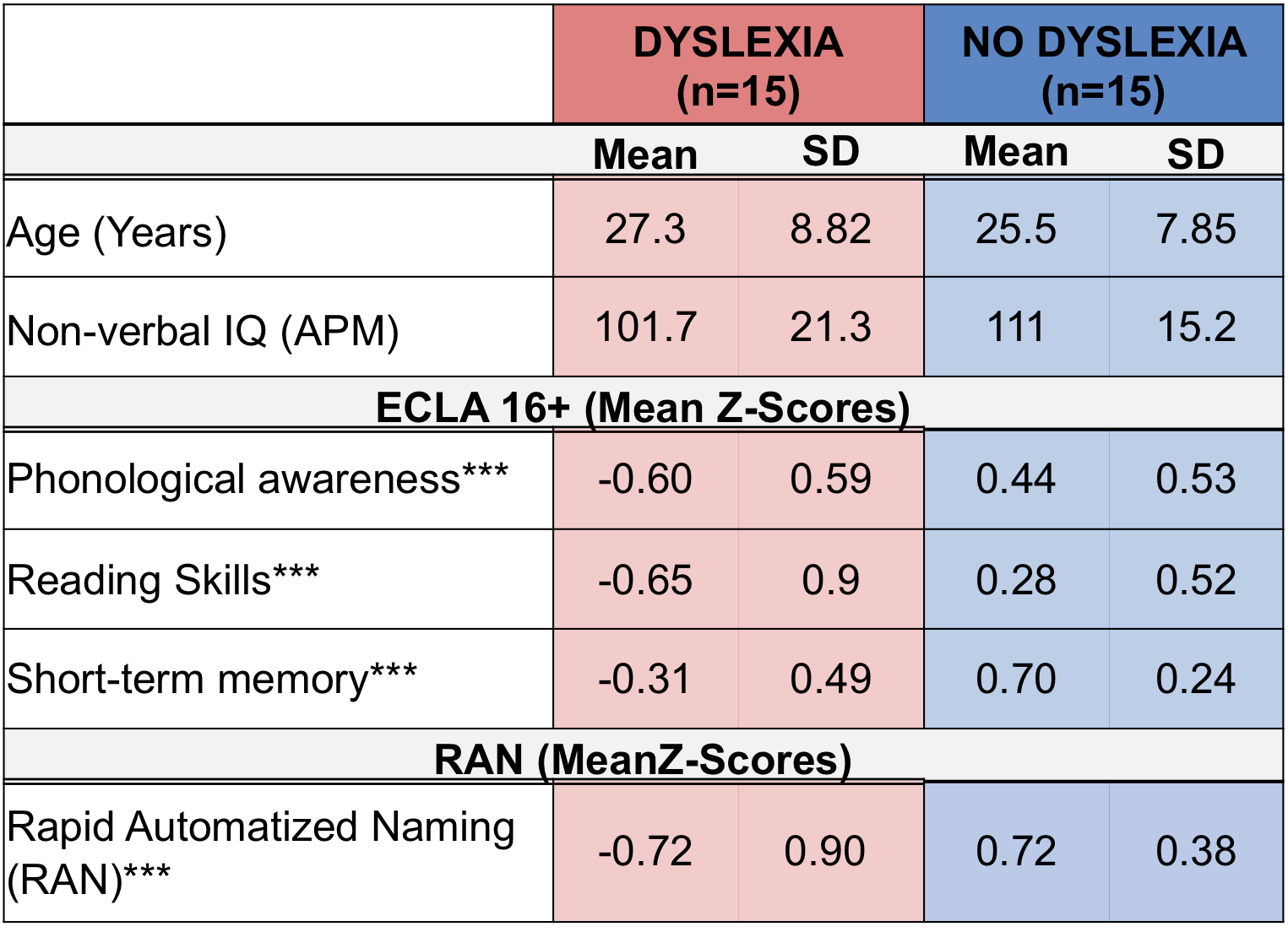

Supplement: S2 Table — Scores in the dyslexia and no-dyslexia groups for the ECLA16+ diagnosis test and the RAN test evaluating phonological awareness, reading skills, and short-term memory. Negative values represent performance below average, positive values performance above average; all values are z-scores. Stars indicates significant differences between the dyslexia and no-dyslexia groups. Significance is denoted with *** for p < 0.001. ECLA16+, Évaluation de Compétences de Lecture chez l'Adulte de plus de 16 ans; RAN, rapid automatized naming. (TIF) [file pbio.3000833.s010.tif]

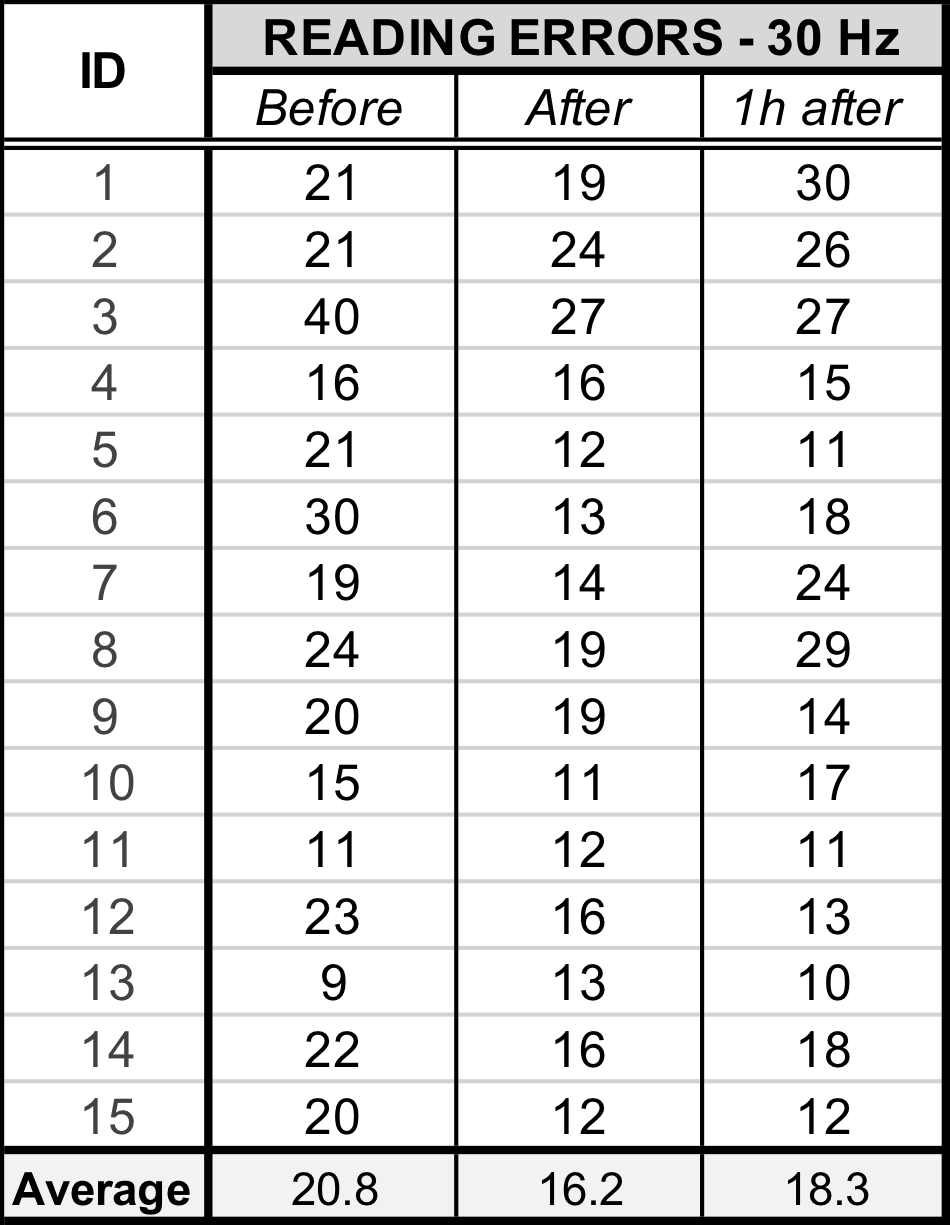

Supplement: S3 Table — Each row corresponds to one participant in the dyslexia group. tACS, transcranial alternating current stimulation. (TIF) [file pbio.3000833.s011.tif]

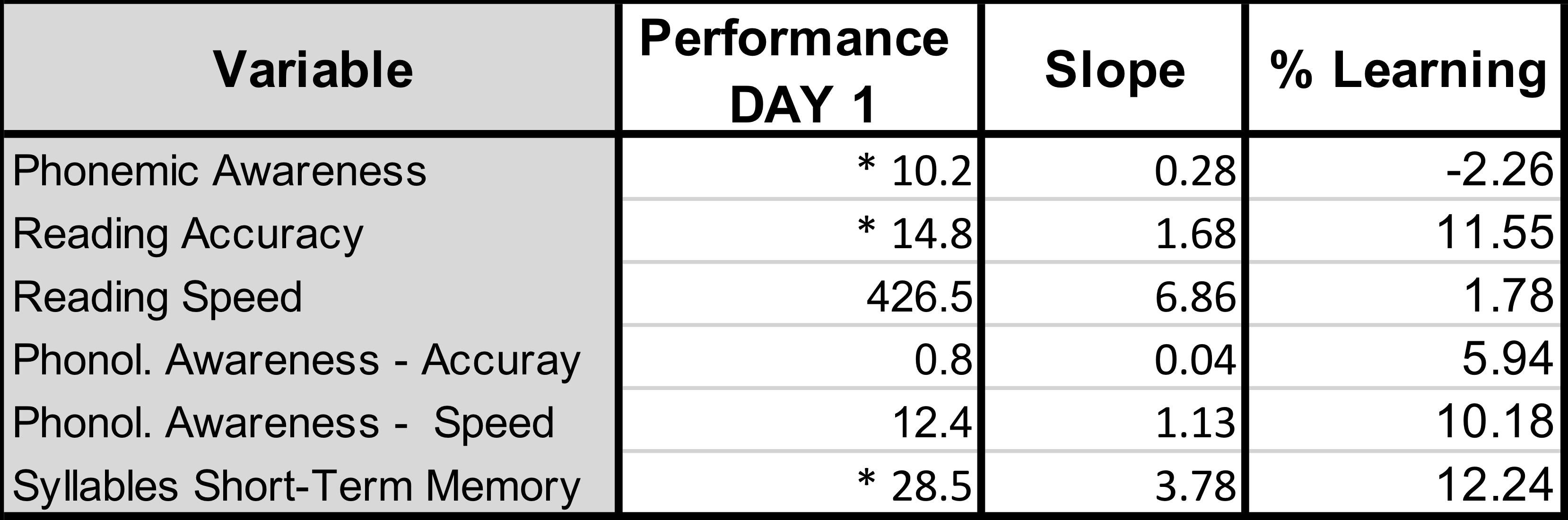

Supplement: S4 Table — Individual slopes were calculated by considering the performance measured “before” stimulation separately for each variable of interest (phoneme, syllables, etc.). The table displays the average performance during the first experimental day (asterisks indicates number of errors instead of absolute performance), the average slopes, and learning rate across participants. The slopes were significantly different from zero (one-sample t test) for all variables except for the Phonemic Awareness index T29 = 0.57, p > 0.05, d = 0.1). They were similar in the 2 groups (dyslexia, no dyslexia) in all variables but phonological awareness response speed (spoonerism test: T28 = 2.39, p < 0.05, d = 0.87, 2-sample unpaired t test). Please note that this index was not analyzed individually but pooled together with the spoonerism accuracy in order to compute a Global Phonological Awareness index. (TIF) [file pbio.3000833.s012.tif]
