# Supplementary material for: Selective enhancement of low-gamma activity by tACS improves phonemic processing and reading accuracy in dyslexia
Source: PLoS Biol. 2020 Sep 8;18(9):e3000833. doi: 10.1371/journal.pbio.3000833 (PMC7478834; doi:10.1371/journal.pbio.3000833)
Supplement: Attachment — Submitted filename: Revision_pointByPoint.docx [file pbio.3000833.s013.docx]

**Reviewer #1:**

Thank you for inviting me to review "Selective enhancement of low-gamma activity by tACS improves phonemic processing and reading accuracy in dyslexia", by Marchesotti, Nicolle, and colleagues (PBIOLOGY-D-20-00231R1). In this manuscript, the authors report that applying 30 Hz tACS to left auditory cortex improves phonemic awareness and reading accuracy in dyslexic subjects immediately following stimulation. This is consistent with a theory of oscillatory auditory processing and lays the groundwork for a possible intervention for a difficult condition with few other treatment options. I therefore expect that these results will be of interest to the diverse readership of PLoS Biology.

In particular, the design and rationale of this brain stimulation experiment is one of the best that I have read. The authors have a clear theoretical reason for choosing 30 Hz tACS plus some experimental support showing a low gamma deficit in the left auditory cortex of dyslexic patients, which they replicate in Figure 1. Possible stimulation confounds are controlled for by blinding and comparing against both sham stimulation and another active stimulus (60 Hz) at the same site. Stimulation has the predicted effect on neural activity, alleviating the 30 Hz EEG power deficit and improving behavioral performance on reading and phonological tasks, but not syllable-based ones. This is a solid careful design for a neurostimulation experiment. My current research is focused on brain stimulation rather than dyslexia, but the linguistic tasks and interpretation also seem appropriate based on what I know and the cited references.

**1- “*That said, I do have a few concerns about the analytic approach. The first is that several analyses use two t-tests or ANOVAs to separately test for an effect (e.g., changes in phonemic awareness before/after tACS) separately in each group (e.g., dyslexic/non-dyslexic). Inferences are then drawn based on which groups did or did not show significant differences. This approach is generally not advisable (see Gelman and Stern, 2006). Instead, the authors should directly test the changes of interest (e.g., does the change in reading accuracy differ after sham/30/60 Hz). This could also be tackled with a mixed-effects model with interactions. I suspect that the theoretically-interesting comparisons will still pan out, but it would be better be careful.”***

**Answer.**

We thank the Reviewer for bringing up the important issue of statistics, which prompted us to improve our statistical approach. We have now considered a different metrics for the main behavioral analysis, consisting in the difference *immediately after* minus *before* tACS*,* and *1h after* minus *before*. As recommended by the Reviewer, this allows for directly testing changes in performance between the two groups and stimulation condition.

We have given careful consideration to the proposal of running a full mixed model ANOVA including groups (dyslexia, no dyslexia) as between-subjects factor and stimulation conditions (sham, 30 Hz, 60 Hz) and time (after, one hour after) as within-subjects factors. However, we finally rejected this option for the following reasons. Our experimental design was built to test the hypothesis that 20 min. of 30 Hz tACS should induce a transient positive effect on phonological processing in the dyslexia group. The design includes several control conditions (sham, 60 Hz, a control group, a delayed measure one hour after tACS) to address the specificity of the effect. If all these controls were to be included in a single fully exploratory model, the results of interest would be diluted and potential interactions extremely difficult to interpret.

To gain specificity and result interpretability, we opted for two statistical analyses where we probe separately the two post-tACS measurements (respectively *immediately after* and *one hour after* tACS), using a repeated measures ANOVA with group (dyslexia, no dyslexia) as between-subjects factor and stimulation condition (sham, 30 Hz, 60 Hz) as within-subject factor. First, we used this approach to investigate immediate effects (*after-before*) in all metrics (phonemic awareness, reading accuracy and speed, global phonological awareness), and then restrict the analysis on the *1h-after* change in those metrics that showed a modulation immediately after the tACS.

We believe this solution a good compromise, as it allows for strict comparability across behavioural measures, which was not the case before. This comes at the expense of directly comparing the two time delays. To deal with this, we included in the revised manuscript a third simple post-hoc analysis, aiming at specifically testing for the dynamics of the behavioral effect in those metrics that showed one. We believe this analysis to be legitimate, but we let the Reviewers decide whether to include it or not.

The new analysis confirms the results presented in the original version of the manuscript, that is an improvement in phonemic awareness and reading accuracy immediately after the 30 Hz tACS, selectively in the dyslexia group. However, the decrease in reading speed no longer reaches statistical significance, as described in the response to point 5.

Results appears in the revised version as follows (pages 5-7, lines 126-190):

“*To directly compare changes in performance following tACS across stimulation conditions (sham, 30 Hz, 60 Hz) and between the two groups (dyslexia, no dyslexia), we considered the between-time measurements difference, i.e. before and after tACS stimulation. We ran a repeated measures ANOVA with group (dyslexia, no dyslexia) as between-subjects factor and stimulation condition (sham, 30 Hz, 60 Hz) as within-subject factor. We first assessed the effect of tACS on phonemic awareness, considering the number of errors in the repeated phonemes at the pseudo-word test. We found a main effect of group (F_1,28_ = 11.4, p < 0.01, η²_p_ = 0.28), stimulation condition (F_2,56_ = 3.16, p = 0.05, η²_p_ = 0.1) and a significant interaction between group and stimulation conditions (F_2,56_ = 3.96, p < 0.05, η²_p_ = 0.12, Fig* ***2A****). Post hoc tests revealed that the improvement after 30 Hz tACS was stronger in the dyslexia than in the control group (T_74.8_ = -4.27, p < 0.001, d = 1.68, FDR), as well as for sham (T_56_ = -2.62, p < 0.05, d = 0.73, FDR) and 60 Hz tACS (T_56_ = -3.64, p < 0.01, d = 0.75, FDR) in the dyslexia group.*

*[…] To address whether phonemic awareness improvement following 30 Hz-tACS stimulation in the dyslexia group could ultimately result in, or be associated with, better reading performance, we extracted and analysed separately changes in reading accuracy (see Language assessment section in the Methods) and speed from the text reading task. With respect to post-stimulation reading accuracy (Fig* ***2C****), we found a main effect of group (F_1,28_ = 4.89, p < 0.05, η²_p_ = 0.09) and of the stimulation condition (F_2,56_ = 3.13, p = 0.051, η²_p_ = 0.1), as well as a marginal group x stimulation interaction (F_2,56_ = 2.5, p = 0.09, η²_p_ = 0.08). Given this trend and the results found for phonemic awareness, we tested for the same pattern in reading accuracy. As for phonemic awareness, the reading accuracy improvement after 30 Hz tACS was stronger in the dyslexia than the control group (T_83.6_ = -3, p < 0.05, d =0.96), and stronger than in the sham condition in the dyslexia group (T_56_ = -3.2, p < 0.05, d =0.67). A similar trend was observed for the 60 Hz tACS condition (T_56_ = -2.37, p = 0.06, d =0.45).*

*[…] Although we did not find any significant change in performance in reading speed, a qualitative analysis of the results (Fig* ***S4-A****) suggests that reading speed was slowed-down after all stimulation conditions, regardless of dyslexia severity. These results could indicate that the phonemic awareness improvement translated into reading accuracy, whereas reading speed was altered by a non-specific tACS effect.*

*Last, we found no effect of tACS in global phonological performance, as assessed by the spoonerism test (Fig* ***S4-B****).*

*Given the specific effectiveness of 30 Hz tACS in enhancing phonemic awareness and reading accuracy in the dyslexia group, we then investigated the persistence of the effects by considering the data acquired 1h after the stimulation. We applied the same statistical approach as for the previous dataset (i.e. difference after-before): we run a repeated measures ANOVA with tACS condition (sham, 30 Hz, 60 Hz) as within-subjects factor and group (dyslexia, no dyslexia) as between-subjects factor considering as dependent variable the performance difference between the 1h after and before conditions. For both measures, results did not reveal any statistically significant results for either the tACS condition, group, or interaction between the two factors (Fig* ***S5****).*

*The decline of the immediate tACS effect was confirmed by a one-way repeated measures ANOVA with time (before, after, 1h after) as within-subjects factor on the data (absolute values) of the dyslexia group for the 30 Hz tACS condition for the phonemic awareness and reading accuracy index. Both analyses revealed a main effect of time (phonemic awareness: F_2,28_ = 7.51, p < 0.01, η²_p_ = 0.35, reading accuracy: F_2,28_ = 4.35, p < 0.05, η²_p_ = 0.23, Fig* ***2D****). Performance increased after tACS for both indexes (phonemic awareness: T_28_ = 3.78, p < 0.01, d = 0.97, reading accuracy: T_28_ = 3.08, p < 0.05, d = 0.79, FDR corrected), and decreased one hour after for the phonemic awareness index only (T_28_ = -2.56, p < 0.05, d = 0.66, FDR corrected)*”.

**2- “*Secondly, Figure 2C/2E and 4C show a single correlation coefficient/line, which is presumably calculated from all of the subjects and meant to show differential improvements in the most severely affected subjects. I think it would be better to add separate lines and coefficients for each group (e.g., by adding red and blue lines/numbers)*. *I would like to rule out an issue where the correlation coefficients just reflect differences in the group means rather than individual subjects. Specifically, the healthy controls will tend to be clustered towards the right (since ECLA 16+ measures reading proficiency) and below the x-axis (since they are already near ceiling per Figure 2); dyslexic patients will be towards the left, but less downward as the ceiling effect is smaller. In a quick simulation, I found that this might be enough to produce some spurious correlation even if there is no effect. This could be ruled out by showing that the correlations hold up within each group (or equivalently, the right patterns of main effect + interactions).”***

**Answer.** The Reviewer’s intuition that the correlation is mainly driven by the group means is correct. However, since there is a continuum from typical reading to dyslexia in the ECLA16+ test, we think that it is informative to present how the 30Hz tACS effect is distributed along this dimension. We now indicate in the text that there was no significant effect within each group for the phonemic awareness index (dyslexia: r = 0.09, p = 0.73, normo-readers: r = 0.36, p = 0.18, page 6, lines 160-161), nor for reading accuracy (dyslexia: r = -0.33, p = 0.23, normo-readers: r = 0.03, p = 0.9, page 7, line 166), and that the between group correlation mainly highlights that while there is a gain in performance when the ECLA16+ score is weak, the effect is rather disruptive when ECLA16+ is high.

Accordingly, we have rephrased sentences referring to this correlation so to avoid strong claims of correlation as follows:

Abstract (page 2, lines 22-24): “*The intervention significantly improved phonological processing and reading accuracy as measured immediately after tACS. The effect occurred selectively for a 30 Hz stimulation in the dyslexia group*”.

Discussion (page 9, lines 249-250): “*Interestingly, the facilitating tACS effect on phonological processing was most pronounced in individuals who had poor reading skills, whereas a slightly disruptive effect was observed in very good readers*”.

Plots relative to these correlation analyses appear now as a figure in the supplementary material (Fig. S3).

**3- “*The mechanistic discussion in Lines 241-258 is perhaps a little speculative, given what we currently (don't) know about tACS after effects. While I agree that some form of plasticity is likely involved, I'm not sure the paper really requires a deep dive into possible molecular mechanisms until they are better understood. On the other hand, Line 259 may undersell their results—an intervention that only helps during or immediately after stimulation could potentially still be a big win for patients with dyslexia.”***

**Answer.** We thank the Reviewer again for raising this cautionary note about the potential underlying molecular mechanisms of tACS intervention on dyslexia. The reason why we elaborated on the issue was to contextualize the present work in a broader context, allowing the reader to connect it to possible molecular accounts. We still think such a contextualization might be interesting for future studies on dyslexia. We would hence prefer to keep the discussion as is, although we can of course remove it if the Reviewer (or the editor) thinks this really detracts from the paper.

We modified lines 285-287 (page 11) as follows:

*“Although a single intervention acutely improved performance, a chronic treatment could be expected to not only durably normalize the phonemic sampling capacity, but also induce large scale modifications of the reading network”.*

**Overall, I think this is a well-designed experiment and only needs minor changes to the analysis, described above. A few more suggestions/trivial errors are listed below.**

---------

Other comments:

**4- *“Line 131 (and others): I found it confusing that the time point factor (before/immediately after/1 hr) was called "session", which could also refer to sham/30/60 Hz stimulation, as it does in the legend of Figure S1. If you do not want to rename the factor, I suggest explicitly defining it around Line 128: "During each day, subjects' language abilities were tested in three sessions: before stimulation, immediately afterwards, and one hour later."***

**Answer.** We thank the Reviewer for the suggestion. We agree that the term session was not ideal to convey the notion of time. We have now renamed throughout the manuscripts and figures this factor into “time”.

**5- *“Line 165-6: "reading speed was slowed-down immediately after all stimulation conditions". The data in Figure S3 suggests things are a little more complicated. Specifically, dyslexic subjects show slowing after both stimulation (but not sham) conditions; non-dyslexics showed slowing after sham and 60 Hz. Some of this is likely due to confounds (subjects are more tired/distractable, etc right after stimulation). This possibility should be tested more directly by comparing the changes in speed across conditions, instead of drawing inferences between which before/after comparisons are/are not significant.”***

**Answer.** By considering behavioural differences *before* and *after* tACS, as reported in the current revision, we did not confirm the slowing down of reading speed presented in the previous version. This is due to the fact that the decrease in reading speed occurred right after the tACS stimulation, for most tACS conditions and in both groups (as indicated by the negative values in the figure below, left). We agree with the Reviewer that this effect might be due to confounds such as fatigue. We now report results on the change *after-before* as follows (page 7, lines 168-170):

*“Although we did not find any significant change in performance in reading speed, a qualitative analysis of the results (Fig* ***S4-A****) suggests that reading speed was slowed-down after all stimulation conditions, regardless of dyslexia severity”.*

**6- *“Line 173: "20 min tACS [is] not sufficient to positively impact speech production". Is there a reason to think it would? If not, I would suggest rephrasing it.”***

**Answer.** We assume that tACS should act by restoring left lateralized low-gamma activity in the auditory cortex and immediate surrounding regions. Although this effect is expected to have an immediate impact on phonemic awareness, it might not readily translate into a reading fluency improvement. An improvement on reading speed would require a period of reorganization of the language network, which is unlikely to occur within 20 min.

As the tACS effect on reading speed is no longer significant with the new analyses we have removed this sentence.

**7- “*Line 389, 392-3: tACS systems vary in how they report stimulation intensity (peak-to-peak, 0 to max, RMS). I believe the system used here reports 0-max, so it might be clearer to report the stimulation intensity as "starting from ±0.8 mA" or "with a maximum intensity of 4 mA (peak to peak).*"**

**Answer.** In the revised version we now refer to peak-to-peak intensities.

**8- “*Line 385: The PISTIM electrodes' inner diameter is 2 cm (so that the total area is πcm²) rather than 1.2 cm reported here (which is the outer radius, but includes the 0.2 mm inactive plastic lip).”***

**Answer.** We thank the Reviewer for catching the error. We now report that the correct contact area is equal to πcm² (page 15, line 421).

**9- “*Line 416: Rescaling between 0 and 1 is probably the right thing to do for the data analysis, but it makes it hard to gauge the practical effect of this intervention. It might be worth slipping an unscaled version of the reading effect size (like 'subjects misread 10 fewer words') into the results or discussion.*”**

**Answer:** We agree with the point raised by the Reviewer and in the revised version of the supplementary material we now provide a table (Table **S3**, see below) reporting the individual raw data for reading errors (used to evaluate reading accuracy) in the dyslexia group at the three *time* measurements during the 30 Hz tACS experimental day. After the 30 Hz tACS, reading errors dropped from 20.8 to 16.2 as compared to the pre-tACS measurement.

***Table S3****. Individual number of reading errors in the dyslexia group at each time measurement during the 30 Hz tACS condition. Each row corresponds to one participant in the dyslexia group.*

**10- *“Line 441: Pearson, rather than Person, correlation.”***

**Answer.** This has been corrected in the text (page 18, line 484).

**11- “*Figure 1 (c. Line 733). Is the brain shown in Panel B one of the participants or an atlas? Either is fine, but it should be indicated in the caption.”***

**Answer.** We now present this information in the caption of Fig. 1 as follows: “*Simulation of the electric field induced by the tACS stimulation using a high-definition 4x1 electrodes configuration,* ***displayed on a default cortical surface***”. We have also added the preprint reference relative to the software employed to obtain the model.

**12- “*Figure 2B (c. Line 747). It is unclear which 'after' condition is meant by the light gray bars. The text hints at 'immediately after', but the color scheme in 1A/1D suggests '1 hr after'.”***

**Answer.** Given the changes in the statistical approach and the visualization of the main analyses, we believe that the revised figure (Figure 2B) is now easier to interpret.

**13- “*Figure 2,4, S2, S (c. Lines 747, 780, 819, 834). It is not clear what the small numbers in Panel 2C, 2D, 4C, S2C, S3B, and S3C indicate.”***

**Answer.** The numbers displayed in the scatter plots refer to the participants ID. We have realized this information was not relevant and removed the numbers in the new version of the plots.

***14-* “*Figure 4 (c. Line 780) It would be nice if the Y axes in Panel C matched (they are close, but not quite the same).”***

**Answer.** We thank the Reviewer for raising this point. It allowed us to discover an inconsistency in the previous analysis, which is now solved. We now show a stronger relationship between the degree of phonemic improvement and the power increase (figure below, panel C-right).

Reference:

Gelman, A., & Stern, H. (2006). The difference between "significant" and "not significant" is not itself statistically significant. The American Statistician, 60(4), 328-331.

**Reviewer #2:**

In the study "Selective enhancement of low-gamma activity by tACS improves phonemic processing and reading accuracy in dyslexia" Marchesotti and colleagues investigated the effect of 20 min 30 Hz tACS, as well as 60 Hz tACS and sham tACS, over the left auditory cortex on phonological processing and reading accuracy in 15 adult dyslexic and 15 healthy control participants.

In result the authors confirm reduced 30 Hz ASSR responses in the left auditory cortex for dyslexic participants compared to healthy controls. Importantly they further show that 30 Hz tACS improved phonemic awareness as well as reading accuracy in the dyslexic but not in the control group from pre to post measure. Furthermore, tACS induced improvements in phonemic awareness and reading accuracy were related to the severity of the dyslexic symptoms across all participants. Finally, 30 Hz tACS increased the 30 Hz ASSR response in dyslexic but not in healthy control participants.

This is a highly relevant study addressing a very interesting and timely topic. On top of this, the manuscript is theoretically well embedded and proportional and concisely written.

However, unfortunately the manuscript in its present form comprises several methodological issues that prevent a publication in PLOS Biology. In sum, the statistical approach is invalid, data smoothing/handling is insufficient explained and the process under investigation remains elusive. Please find below a more detailed list of concerns/problems/questions that might help improving the manuscript.

Statistics:

**1- “*In its present form the statistic approach looks like "cherry-picking" in it selects only a subset of available data and statistical tests that support their claims. In their experimental design the authors included 2 (groups) by 3 (tACS conditions [30Hz, 60Hz, sham]) by 3 (measurement [pre, after, 1h after]) factors but none of the performed analyses does include all of them. In contrast the authors performed 2 (group) x 3 (measurement) ANOVAs separately for each stimulation condition for behavioral data 1 (phonemic awareness) BUT 3 (pre, after, 1h after) x 3 (30Hz, 60Hz, sham) ANOVAs separately for each group for behavioral data 2 -4 (accuracy, speed text reading, spoonerism) without valid justification.”***

**Answer.** We thank the Reviewer for pointing out these discrepancies, which rejoin Reviewer 1’s criticisms. We now present a consistent statistical approach, as detailed below.

We have now considered a different metrics for the main behavioral analysis, consisting in the difference *immediately after* minus *before* tACS*,* and *1h after* minus *before*. This allows for directly testing changes in performance between the two groups and stimulation condition.

We have given careful consideration to the proposal of running a full mixed model ANOVA including groups (dyslexia, no dyslexia) as between-subjects factor and stimulation conditions (sham, 30 Hz, 60 Hz) and time (after, one hour after) as within-subjects factors. However, we finally rejected this option for the following reasons. Our experimental design was built to test the hypothesis that 20 min. of 30 Hz tACS should induce a transient positive effect on phonological processing in the dyslexia group. The design includes several control conditions (sham, 60 Hz, a control group, a delayed measure one hour after tACS) to address the specificity of the effect. If all these controls were to be included in a single fully exploratory model, the results of interest would be diluted and potential interactions extremely difficult to interpret.

To gain specificity and result interpretability, we opted for two statistical analyses where we probe separately the two post-tACS measurements (respectively *immediately after* and *one hour after* tACS), using a repeated measures ANOVA with group (dyslexia, no dyslexia) as between-subjects factor and stimulation condition (sham, 30 Hz, 60 Hz) as within-subject factor. First, we used this approach to investigate immediate effects (*after-before*) in all metrics (phonemic awareness, reading accuracy and speed, global phonological awareness), and then restrict the analysis on the *1h-after* change in those metrics that showed a modulation immediately after the tACS.

We believe this solution a good compromise, as it allows for strict comparability across behavioural measures, which was not the case before. This comes at the expense of directly comparing the two time delays. To deal with this, we included in the revised manuscript a third simple post-hoc analysis, aiming at specifically testing for the dynamics of the behavioral effect in those metrics that showed one. We believe this analysis to be legitimate, but we let the Reviewers decide whether to include it or not.

The new analysis confirms the results presented in the original version of the manuscript, that is an improvement in phonemic awareness and reading accuracy immediately after the 30 Hz tACS, selectively in the dyslexia group. However, the decrease in reading speed no longer reaches statistical significance, as described in the response to Reviewer 1’s point 5.

For the changes to the manuscript please refer to our response to Reviewer 1’s point 1.

**2- “*The correlations performed include all participants but the results are interpreted for the dyslexic patients only. Is there a valid correlation in patients only or does the correlation mainly represent the group differences?”***

**Answer.** This issue has also been raised by Reviewer 1 in point 2. Indeed, the correlation is mainly driven by the group means. However, since there is a continuum from typical reading to dyslexia in the ECLA16+ test, we think that it is informative to present how the 30Hz tACS effect is distributed along this dimension. We now indicate in the text that there was no significant effect within each group for the phonemic awareness index (dyslexia: r = 0.09, p = 0.73, normo-readers: r = 0.36, p = 0.18, page 6, lines 160-161), nor for reading accuracy (dyslexia: r = -0.33, p = 0.23, normo-readers: r = 0.03, p = 0.9, page 7, line 166), and that the between group correlation mainly highlights that while there is a gain in performance when the ECLA16+ score is weak, the effect is rather disruptive when ECLA16+ is high.

Accordingly, we have rephrased sentences referring to this correlation so to avoid strong claims of correlation as follows:

Abstract (page 2, lines 22-24): “*The intervention significantly improved phonological processing and reading accuracy as measured immediately after tACS. The effect occurred selectively for a 30 Hz stimulation in the dyslexia group*”.

Discussion (page 9, lines 249-250): “*Interestingly, the facilitating tACS effect on phonological processing was most pronounced in individuals who had poor reading skills, whereas a slightly disruptive effect was observed in very good readers*”.

Plots relative to these correlation analyses appear now as a figure in the supplementary material (Fig. S3).

**3- “*Analogously, when "confirming" the 30 Hz response deficit (ASSR) in the left AC a 2 (group) x 2 (hemisphere) ANOVA should have been performed resulting in a significant interaction (not done). Even though I agree that in this case there is a strong and valid a-priori hypothesis allowing for direct testing.”***

**Answer.** The Reviewer is right in the sense that an ANOVA with both *hemisphere* (left/right) and *group* (dyslexia/no dyslexia) would be correct in the absence of a strong a priori hypothesis. As pointed out by the Reviewer, our experimental design was informed by previous work, and interpreting any tACS effect required first to confirm reduced activity in the left auditory cortex in the dyslexia group relative to normo-readers [1]. We now present the requested interaction which is not significant (F_1,27_ = 2.05, p = 0.16, η²_p_ = 0.07), but also the direct effect regarding the left auditory cortex only as it is a strong a priori of the study (page 5, lines 117-121).

**4- “*But why are the pre-to-post effects of 30 Hz tACS on the 30 Hz ASSR tested separately for each group and each stimulation condition?”***

**Answer.** The aim of this analysis was not to explore group differences (which can be more accurately assessed with analysis in the source space), but only to show the specificity of 30 Hz tACS on the EEG power in response to sounds modulated with the same stimulation frequency (and not with other ASSR frequencies).

**5- “*Finally, while the authors eventually performed a 2 (pre, post) x 2 (hemisphere)) ANOVA for the ROI analysis (only for 30 Hz tACS, separately for each group) they continue to invalidly post-hoc test pre-to-post changes in the left and right AC without a significant interaction of both parameters.”***

**Answer.** The choice of testing separately the 30 Hz tACS effect in the two auditory cortices in the dyslexia group is motivated by the a priori hypothesis that the stimulation should impact the left hemisphere. It is hence of primary importance to show that the tACS effect had a local effect. We now make explicit this is also a strong a priori in the main text (page 8, lines 206-211) as follows:

“*In the auditory cortex, we found a significant power increase after 30-Hz tACS (main effect of time, F_1,14_ = 13.71, p < 0.01, η²_p_ = 0.49, Fig 4A). We then tested whether the 30 Hz tACS applied over the left auditory cortex had a local impact on the neural activity in this region. We found a significant increase in oscillatory response in the left auditory cortex (T_14_ = 3.4, p < 0.05, d = 0.88, FDR corrected, Fig 4A). The effect was not present in the right AC (T_14_ = 1.6, p > 0.05, d = 0.42, FDR corrected, Fig 4A), even though there was no time x hemisphere interaction.*”

**6- “*Finally, EEG data for the "1h-after" measure are completely ignored.*”**

**Answer.** As correctly noted by the Reviewer, the *1 hour* post-tACS EEG data are not reported. The EEG data acquired *one hour after* tACS condition were characterized by a poor signal quality due to the duration of the acquisition (approximately 4 hours after the EEG set-up). We have now included this information in the revised version of the manuscript (page 18, lines 504-507) as follows:

“*EEG data acquired one hour after the tACS conditions had poor signal quality due to the duration of the acquisition (approximately 4 hours after the EEG set-up). In a few cases we even had to interrupt the EEG recording session. For this reason, the one hour after EEG datasets have not been taken into consideration*”.

Note that the EEG analysis was intended to 1- confirm the 30 Hz deficit in the dyslexia group, 2- assess whether potential tACS effect could be accounted for by a change in local neural activity. We believe that the fulfilment of these goals is not precluded by the unavailability of the “one hour after” EEG dataset.

We hope that the Reviewer will agree with us on this point.

**Data processing:**

**7- “*Do the authors really assume that the results from a separate pilot group performing the behavioral test-battery 3 times are comparable to the data of the experimental groups performing the behavioral test-battery NINE times?”***

**Answer.** We ran the pilot study to assess that the three sub-groups of tests (one for each time measurement, i.e. *before*, *after* and *1h after*) were of equal difficulty, and to assess potential learning effects over one day of experimentation. The pilot hence followed the same timing as the experimental day. Statistical analysis on the pilot data did not reveal any significant difference across the three measurements.

As for the main experiment, participants performed the same sub-test three times, once per experimental day. To account for potential learning effects occurring across experimental days, we applied the regression approach described in the next point.

We now added more detailed information concerning the pilot, and changed the text (pages 13-14, lines 361-376) as follows:

*“The development of a custom-designed solution was necessary to ensure the diversity of linguistic material across time measurements (before, after, 1h after) and minimise learning effects that may occur over repeated measurements within the same experimental day. All versions of each of the three sub-group of tests (one for each measurement, before, after and 1h after) had similar phonological and syntactic features, as well as the same lexical frequency.*

*To assess equal difficulty in all three tests and exclude learning effects within one day, we run a pilot study in 28 normo-readers, different from those included in the main experiment. Participants performed the three sub-groups of language tests in the same order and with the same timing as in the experimental paradigm. As statistical tests did not show a significant difference across measurements, we assumed that there was no difference in terms of difficulty within the test material, and no detectable learning effect over three consecutive measurements (one-way repeated measures ANOVA). This triple battery of tests was then used during each of the three experimental days (one for each tACS condition), leading to a total of three repetitions per sub-test. To account for potential learning effects occurring over the three experimental days, we regressed out the slope relative to the improvement as described in the paragraph Statistical analysis of language tests”.*

**8- “*The information given for the performed correction of the observed repetition effects needs to be improved. How strong was the repetition effect and how was this "corrected"?”***

**Answer.** The correction was performed as follows: univariate linear regression was performed separately for each variable of interest, e.g. phonemic awareness, on the “before” sessions across all three experimental days in the order of occurrence. The slope of the linear regression was then used to subtract any trend, i.e. repetition effect, from all performance values, i.e. “before”, “after” and “one hour after” measurements of a given experimental day.

We now present as supplementary material (Table S4) a quantitative evaluation of the learning effect, including the raw performance during the first experimental day (the asterisk indicates the number of errors) averaged across participants, the average slope value and learning rate. This was obtained by calculating the percentage of change with respect to the performance recorded during the first experimental day.

In addition, we tested for each variable separately, whether the slope values were significantly different from zero using a one-sample t-test, and found that this was the case for all variables except for the Phonemic Awareness index T_29_ = 0.57, p > 0.05, d = 0.1). We also tested for differences between groups (independent t-test) and found one only for spoonerism response speed (phonological awareness-speed: T_28_ = 2.39, p < 0.05, d = 0.87). Please note that this index was not analyzed individually but pooled together with spoonerism accuracy in order to compute a Global Phonological Awareness index. These statistical analyses are also presented in the legend of Table S4.

**9-** **“*Does any statistical result survive if the test would have been performed without reducing the variance of the behavioral data between subjects? Why wasn't this correction applied to the ECLA scores as well?”***

**Answer.** Being a linear operation, normalizing the data (0-1 range) between subjects did not affect the statistical analyses. This was done only to simplify interpretation and visualisation of the results, given the marked difference in the range of the tested variables. Note that rescaling was necessary for the computation of a global phonological awareness index, obtained by pooling together spoonerism accuracy and speed.

The ECLA-16+ scores represent a z-score calculated from a reference population of 311 individuals recruited to develop this diagnostic test and are based on the mean and standard deviation of each individual sub-test composing the ECLA-16+ index (<http://www.cognisciences.com/accueil/outils/article/ecla-16>, [2]). The ECLA16+ values have therefore to be considered as already standardised (the more negative the more impaired).

**10-** **“*How many different items have been presented during each of the 9 tests for the pseudo-word repetition task? Since the task produced the main behavioral effect of the present study, this information seems essential. The manuscript indicates that the same 30 items were presented each time? If this is true, there needs to be a large repetition effect, especially since several participants showed an - admittedly non-verbal - IQ of >120.”***

**Answer.** There were 30 pseudo-words for each sub-test (90 items in total) and the same sub-tests were used for each experimental day in a different order. In the revised version of the manuscript we modified the *Pseudo-word repetition* section to clarify this point as follows (page 14, lines 379-381):

“*A total of 30 pseudo-words were included in the test: these were different within each sub-test and between the three time measurements (i.e. before, after and 1h after tACS), leading to a total of 90 items. Each pseudo-word consisted of 4 to 8 syllables*”.

**Further:**

**11- “*The EEG positions seem to belong to a 128 channel EEG recording cap and might be referenced accordingly.”***

**Answer.** EEG recordings were performed using a 64-channels cap, with stimulation electrodes placed in between recording channels, and arranged in correspondence of the position indicated in the manuscript (i.e. TTP7h, FTT9h, FCC5h, CPP5h, TPP9h). For clarity, we have now specified this information in the

“Transcranial alternating current stimulation” section of the Methods (page 15, line 422): *“[..] placed over the left auditory cortex and integrated into the* ***64-channels*** *EEG cap to ensure invariant position between participants*”.

**12- “*There is no "anode" or "cathode" during tACS.”***

**Answer**. We thank the Reviewer for pointing out this error, which is now corrected in the manuscript (page 16, lines 425-426).

**13- “*The information about the frequency of stimulation (30Hz or 60 Hz, or separately for both) for the threshold measurement is missing. What was the mean stimulation intensity?*”**

**Answer.** In the revision we provide more detailed information about the stimulation intensity as follows (page 16, lines 432-433): “*The average peak-to-peak stimulation intensity across participants was 1.1 mA for the 30 Hz tACS and 1.2 mA for the 60 Hz condition*”.

**14- “*The stimulation regime was not blinded neither for the experimenter nor for the participants in the end.”***

**Answer.** We understand the Reviewer’s question. As reported in the original version of the manuscript, the stimulation regime was blinded for the participants (“*We conducted this neuromodulation study in a single-blind way in thirty adults*”, page 4, line 82 of the original manuscript) and the validity of the single-blinded approach was assessed by reporting the post-intervention debrief (page 15, lines 399-402 of the original manuscript). We realized that our wording was somewhat confusing, and modified the text as follows (page 16, lines 439-442):

“*Overall, participants’ reports indicate that they could not tell whether they received real or sham tACS: out of 30 participants, only 7 correctly suspected not having received a stimulation during the sham condition, while 18 participants did not suspect any sham tACS and believed having received electrical stimulation over the three experimental days*”.

**Conceptually:**

**15- “*Which process is intended to be manipulated by tACS? Is the 30Hz ASSR deficit related to a PERCEPTUAL impairment?*”**

**Answer.** This is a key point of the study. We thank the Reviewer for raising it and allowing us for further clarification. Lehongre et al. suggested that a 30Hz deficit in left AC could possibly account for different behavioural markers of dyslexia (phonological awareness, rapid naming, verbal memory, [1]). In Lehongre et al.’s study, activity in the left planum temporale in normo-readers showed a positive correlation with phonological skills, whereas this relationship was absent in individuals with dyslexia. However causality cannot be established on the basis of mere correlations. The purpose of the present study was precisely to use tACS to challenge causality between 30Hz deficit and a perceptual impairment. The hypothesis is that a deficit in the left dominance of 30Hz activity could selectively impair the encoding of phonemic cues in the left hemisphere ([1], see also [3] for a theoretical account).

**16- “*There seems to be also inconsistency with respect to the hypothesis of altered gamma oscillations in dyslexia. While the authors initially mention a dysfunctional (to high) sampling rate as the cause of dyslexia they eventually modulate the amplitude of low-gamma oscillations.”***

**Answer.** This again an interesting remark that allows us to further clarify the hypothesis tested in this study. Previous work shows that in individuals with dyslexia activity is lower at ~30Hz in the left auditory cortex but enhanced above 40 Hz mostly in the right auditory cortex [1]. Theoretical work suggests that phonemic encoding could be underpinned by 30 Hz activity [4,5] while higher frequency activity might have an impact on working memory [1]. In this study we chose to restore 30Hz neural activity and address whether it could positively alter phonemic processing. Modulating higher frequency neural activity could be the object of another study, in which we would assess working memory, rather than phonology.

**17- “*How should an improvement in phonemic awareness instantaneously result in an improved reading performance?*”**

**Answer.** We did not expect a direct and immediate improvement of reading, but possibly an indirect one via the restoration of phonemic encoding. Adult readers do not tap much into phonemic awareness while reading, even though this is a key component of reading acquisition. That only reading *accuracy* (and not *speed*) improved immediately after tACS is compatible with this hypothesis, as it suggests an immediate impact of phonemic format without the network having had time to fully reorganize to produce a more fluent reading.

**Reviewer #3:**

The present manuscript examines whether transcranial alternating current stimulation (tACS) at 30 Hz improves phonological processing in dyslexic individuals. The notion underlying the study is the previous demonstration that (1) dyslexic adults show a deficit in oscillatory activity at a frequency corresponding to the phonetic rate in human speech (~30 Hz) and that (2) tACS can boost oscillatory activity.

Indeed, the authors report that (1) dyslexic individuals show a reduced ("auditory steady-state") response (ASSR) to amplitude-modulated sounds at 30 Hz (as compared to healthy individuals); (2) this response is selectively boosted (i.e. only at 30 Hz) after 30-Hz tACS; (3) the enhancement in 30-Hz ASSR goes along with improvements in phonological processing; (4) this effect is not present after sham stimulation or stimulation at 60 Hz.

I can see no major flaw in this manuscript. The manuscript is structured very clearly and logically. Experimental design and analyses are sound. Results are exciting given that they reveal potential treatment for dyslexic individuals in the future. I do have a few comments which I hope can be used to improve the manuscript even further.

**1- “*It is unclear what participants are instructed to do during the stimulation. Did they attend the electric stimulation passively, without sensory stimulation?*”**

**Answer.** We thank the Reviewer for prompting us to provide more details about the task. We have now added a sentence in the Methods section of the revised manuscript clarifying this point as follows *(page 16, lines 436-438)*:

*“During the tACS, as for the EEG recording, participants sat comfortably in front of a screen, watching a muted video of their choice, but without receiving any auditory stimulation”.*

**2- “*The current applied during tCS modulates membrane potentials only by a very small amount (~0.3 mV) and therefore needs to interact with ongoing neural activity to produce a measurable outcome (Stagg & Nitsche, 2011). If auditory cortices are inactive during stimulation (i.e. ongoing neural activity is reduced), I wonder how the authors can explain the efficacy of the stimulation - could they comment on this, please.*”**

**Answer.** This is an interesting question. As participants watched a silent movie during the tACS stimulation, we indeed expected reduced but not null neural activity in auditory cortices [6,7]. On the other hand, resting state does not imply a complete absence of oscillatory activity (see e.g. [8,9]), although it might indeed be weak.

Concerning the tACS effect on oscillatory activity, previous studies indicate that oscillation power can increase after tACS only when baseline oscillatory activity is low [10,11]. The authors propose that this could be due to a ceiling effect in that the oscillation power in a high/normal power state cannot be further enhanced, at least with the well tolerated current intensities. If controls indeed had fully functional oscillations, it could explain that we observed a 30 Hz activity enhancement in subjects with dyslexia (who had reduced 30 Hz activity to start with) but not in controls. However, at the behavioural level, the tACS effect in controls is slightly disruptive (see Figure 2), which calls for yet another interpretation that we cannot provide at this stage.

Please also note that transcranial alternating current stimulation during rest are common practice in clinical contexts (dystonia [12], tremor suppression [13], major depressive disorder [14] and auditory hallucinations [15]).

**3-** **“*The authors frame their observed effect as a demonstration of tACS-induced manipulation of oscillatory activity at 30 Hz. However, they simply show that 30-Hz tACS modulates EEG responses to amplitude-modulated sounds at 30 Hz (i.e. 30-Hz ASSR). There is no demonstration that genuine oscillatory activity is modulated (e.g., it is not shown that resting-state oscillations at 30 Hz are altered, nor that these are reduced in dyslexic individuals). It is possible that tACS merely modulates neurons that respond preferentially to a stimulus frequency corresponding to that of the applied tACS. I therefore recommend to tune down the claim that oscillations are involved; I do not think it needs to be very prominent anyway (if tACS can be used to boost phonological processing, it is only of secondary importance if this entails a manipulation of neural oscillations).”***

**Answer.** This is a key remark. Neural oscillations are not necessarily defined by the fact that they are ongoing at rest. Resting biophysical oscillations can be “weak”, in which case they mostly appear when there is an input [16,17]. ASSRs are known to reflect the ability of the auditory system to enter in a form of resonance with a sound’s rhythmicity, which is different from a mere response to a sound (evoked activity). We therefore remain convinced that ASSRs probe auditory cortex induced resonance properties, i.e. network latent/weak oscillatory properties.

**4- “*A paper is cited entitled "Transcranial electrical stimulation improves phoneme processing in developmental dyslexia". I wonder whether this paper needs to be given more attention, given the similarity of the research question. Could the authors please elaborate on the difference between their study and the cited one.*”**

**Answer.** The Rufener et al. paper is an important reference indeed [18]. However, the authors used 40Hz tACS and transcranial random noise stimulation (tRNS) in adults and children with dyslexia, but did not analyse EEG in the frequency domain. The absence of results on the oscillatory activity prevented the authors from exploring any direct causality between gamma oscillations and phoneme processing. Furthermore, the authors did not investigate potential relationships between behavioural effect and neural activity, as in the present work.

Accordingly we wrote (page 9, lines 240-243): *“However, as these studies have either used transcranial direct current stimulation (tDCS) that do not target specific brain rhythms or tACS but without showing a specific effect on oscillatory activity, the exact neural bases of performance enhancement could not be specified”.*

**5- “*The authors show 30-Hz ASSR aftereffects only for electrode FCz; It would be interesting to see the effect for the whole topography, as tACS might affect neural activity measured at electrodes other than that where we see the strongest ASSR.*”**

**Answer.** We also thought this could be the case. We now present as supplementary material (Figure S6) the topography in the dyslexia group showing t-values obtained by comparing (two-tailed paired-sample t-tests) the EEG data recorded before and after 30 Hz tACS in the 30 Hz ASSR condition.

As appears in the figure below, only FCz showed a significant positive effect, while one frontal electrode Fp2, showed a negative one.

**Fig S6**. *Difference at the topographical level in power response to auditory stimuli at 30 Hz measured before vs immediately after the 30 Hz tACS in the dyslexia group. The topography displays t-values for each of the 64 channels obtained through two-tailed paired-samples t-test. Positive values indicate a stronger response after the 30 Hz tACS; black dots highlight electrodes with p < 0.05.*

**References**

1.	Lehongre K, Ramus F, Villiermet N, Schwartz D, Giraud AL. Altered low-gamma sampling in auditory cortex accounts for the three main facets of dyslexia. Neuron. 2011;72: 1080–1090. doi:10.1016/j.neuron.2011.11.002

2.	Gola-Asmussen C, Lequette C, Pouget G, Rouyet C, Zorman M. (Reading skills assessment for adults over age 16) ECLAT 16+ : Evaluation des compétences de lecture chez l’adulte de plus de 16 ans. 2010.

3.	Hyafil A, Giraud AL, Fontolan L, Gutkin B. Neural Cross-Frequency Coupling: Connecting Architectures, Mechanisms, and Functions. Trends Neurosci. 2015;38: 725–740. doi:10.1016/j.tins.2015.09.001

4.	Hyafil A, Fontolan L, Kabdebon C, Gutkin B, Giraud AL. Speech encoding by coupled cortical theta and gamma oscillations. Elife. 2015;4: 1–45. doi:10.7554/eLife.06213

5.	Hovsepyan S, Olasagasti I, Giraud A-L. Combining predictive coding with neural oscillations optimizes on-line speech processing. bioRxiv. 2018; 477588. doi:10.1101/477588

6.	Arnal LH, Morillon B, Kell CA, Giraud A-LL. Dual neural routing of visual facilitation in speech processing. J Neurosci. 2009;29: 13445–13453. doi:10.1523/JNEUROSCI.3194-09.2009

7.	Mégevand P, Mercier MR, Groppe DM, Golumbic EZ, Mesgarani N, Beauchamp MS, et al. Phase resetting in human auditory cortex to visual speech. bioRxiv. 2020; 405597. doi:10.1101/405597

8.	Lakatos P, Shah AS, Knuth KH, Ulbert I, Karmos G, Schroeder CE. An oscillatory hierarchy controlling neuronal excitability and stimulus processing in the auditory cortex. J Neurophysiol. 2005;94: 1904–1911. doi:10.1152/jn.00263.2005

9.	Giraud AL, Kleinschmidt A, Poeppel D, Lund TE, Frackowiak RSJ, Laufs H. Endogenous Cortical Rhythms Determine Cerebral Specialization for Speech Perception and Production. Neuron. 2007;56: 1127–1134. doi:10.1016/j.neuron.2007.09.038

10.	Neuling T, Rach S, Herrmann CS. Orchestrating neuronal networks: sustained after-effects of transcranial alternating current stimulation depend upon brain states. Front Hum Neurosci. 2013;7: 1–12. doi:10.3389/fnhum.2013.00161

11.	Alagapan S, Schmidt SL, Lefebvre J, Hadar E, Shin HW, Frӧhlich F. Modulation of Cortical Oscillations by Low-Frequency Direct Cortical Stimulation Is State-Dependent. PLoS Biol. 2016;14: 1–21. doi:10.1371/journal.pbio.1002424

12.	Angelakis E, Liouta E, Andreadis N, Leonardos A, Ktonas P, Stavrinou LC, et al. Transcranial alternating current stimulation reduces symptoms in intractable idiopathic cervical dystonia: A case study. Neurosci Lett. 2013;533: 39–43. doi:10.1016/j.neulet.2012.11.007

13.	Brittain JS, Probert-Smith P, Aziz TZ, Brown P. Tremor suppression by rhythmic transcranial current stimulation. Curr Biol. 2013;23: 436–440. doi:10.1016/j.cub.2013.01.068

14.	Alexander ML, Alagapan S, Lugo CE, Mellin JM, Lustenberger C, Rubinow DR, et al. Double-blind, randomized pilot clinical trial targeting alpha oscillations with transcranial alternating current stimulation (tACS) for the treatment of major depressive disorder (MDD). Transl Psychiatry. 2019;9. doi:10.1038/s41398-019-0439-0

15.	Ahn S, Mellin JM, Alagapan S, Alexander ML, Gilmore JH, Jarskog LF, et al. Targeting reduced neural oscillations in patients with schizophrenia by transcranial alternating current stimulation. Neuroimage. 2019;186: 126–136. doi:10.1016/j.neuroimage.2018.10.056

16.	Börgers C, Epstein S, Kopell NJ. Background gamma rhythmicity and attention in cortical local circuits: A computational study. Proc Natl Acad Sci U S A. 2005;102: 7002–7007. doi:10.1073/pnas.0502366102

17.	Giraud A-L. Oscillations for all A commentary on Meyer, Sun & Martin (2020). Lang Cogn Neurosci. 2020;0: 1–8. doi:10.1080/23273798.2020.1764990

18.	Rufener K, Krauel K, Meyer M, Heinze H-J, Zaehle T. Transcranial electrical stimulation improves phoneme processing in developmental dyslexia. Brain Stimul. 2019. doi:10.1016/j.brs.2019.02.007
