# Supplementary material for: Selective enhancement of low-gamma activity by tACS improves phonemic processing and reading accuracy in dyslexia
Source: PLoS Biol. 2020 Sep 8;18(9):e3000833. doi: 10.1371/journal.pbio.3000833 (PMC7478834; doi:10.1371/journal.pbio.3000833)
Supplement: Attachment — Submitted filename: PLoS_R2_PointByPoint_final.docx [file pbio.3000833.s014.docx]

**Reviewer #1:**

**Thank you for inviting me to re-review "Selective enhancement of low-gamma activity by tACS improves phonemic processing and reading accuracy in dyslexia" by Marchesotti, Nicolle, and colleagues (PBIOLOGY-D-20-00231R2). As in my initial review, I found the paper to be an interesting and well-written manuscript. The Reviewers' comments and edits have largely addressed the questions I raised in the last round, and the manuscript now seems ready for publication in PLoS Biology.**

**I have a few final suggestions, but all of them are completely optional.**

1- **In the interests of transparency, I would prefer that both the pooled data and separate dyslexic/control correlations be reported in Figure 4c (maybe with smaller, dashed lines). It's up the reviewers.**

**Answer:** we thank the reviewer for providing us these additional suggestions, that we have now integrated in the revised version of the manuscript. We have modified Figure 4C and Figure S3 so to include within group regression lines (see figures below), and modified the figures caption accordingly.

**Fig. 4** […] The relation between 30 Hz power gain in left auditory cortex and behavioral variables was considered across groups *(solid grey lines).* Changes in power correlated negatively with dyslexia severity (**C**-left, r = -0.46, p= 0.01), and positively (trend) with ~~the~~ phonemic improvement (**C**-right, r = 0.36, p = 0.06). *Within group trend lines are displayed with dashed lines (red: dyslexia group, r = -0.15, p = 0.59 for ECLA, r = 0.05, p = 0.85 for phonemic awareness, blue: control group, r = 0.35, p = 0.22 for ECLA, r = -0.036, p = 0.9 for phonemic awareness).*

**Fig. S3. Relationship between language skills and behavioural improvement.** Changes in performance occurring *after* ~~the~~ 30 Hz tACS (difference *after-before*) for both phonemic awareness (**A,** *r = -0.48, p = 0.007*) and reading accuracy (**B**, *r = -0.5, p = 0.005*) are negatively related to language skills (ECLA16+, higher values indicate better reading skills) *across groups (solid grey lines).* *Within group trend lines are displayed with dashed lines (red: dyslexia group, r = 0.09, p = 0.73 for phonemic awareness, r = -0.32, p = 0.23 for reading accuracy; blue: control group, r = 0.36, p = 0.18 for phonemic awareness, r = 0.034, p = 0.9 for reading accuracy).*

2- **The caption on Figure 1B could be revised to indicate that it's a single individual's brain. It might also be useful to include the contralateral hemisphere (or say in the text that no fields above X were detected), as effects on both hemispheres are discussed in the paper.**

**Answer:** This is an interesting remark. We have now added as supplementary material, a figure displaying the electric field in the right hemisphere, as well as a coronal section including its peak value (see figure below). This image shows that the electric field in the hemisphere contralateral to the stimulation site is can be estimated to zero.

Furthermore, to include information concerning the head model used to display the electric field, we have modified the legend in Figure 1B as follows:

*“* […] (B). Simulation of the electric field induced by tACS using a high-definition 4x1 electrodes configuration, displayed on *a head model* *from an individual subject* ~~a default cortical surface~~. The model has been obtained with the freeware softwares SimNIBs, [81] and gmsh ([www.gmsh.info](http://www.gmsh.info))*. The electric field elicited by the tACS can be observed selectively the left hemisphere, more prominently on auditory brain regions, while in the contralateral hemisphere the resultant electric field can be estimated to zero (see Fig. S8).”*

***Fig. S8.*** *Simulated electric field induced by the 4x1 high-definition tACS applied to the left hemisphere (see Fig.1), displayed on an individual head model. Results show null electric field in the right hemisphere, contralateral to the stimulation site. The coronal view at the peak strength show focal activity over left auditory regions.*

3- **In the responses to Reviewers 1 and 2, the 1hr time point is described as a control. Different papers report a wide range of tACS after-effects, from nearly zero to few surprising reports suggesting that a single session produces changes detectable 3-4 hours later, so I am a little skeptical that it is a valuable control. However, if it's what the authors intend, that point could be made more strongly in the paper—I read it more as an interesting follow-up question.**

**Answer:** we thank the reviewer for this comment and we agree that the *1h after* recording session is not a proper “control” condition, as whether the effect lasts or not remains a matter of exploration. We have modified the text as follows:

Lines 81-84, page 4: “*To characterize the effect, the design included several other conditions (a sham stimulation, a 60 Hz tACS condition, a control group, and a delayed measure one hour after tACS where the effects were expected to have largely disappeared).*”

Lines 95-101, page 4: “ASSRs and behavioral tests were performed *before*, *immediately after* and *one hour after* 20 minutes of focal tACS (<= 2mA stimulation) over left auditory cortex, a stimulation duration that is argued to be effective while minimizing potential side effects [32]. *The one hour after tACS was included to both evaluate and control for potential long-term effect of the stimulation.* The specificity of the 30 Hz intervention was *further* controlled using two within-subject conditions: an active control stimulation at 60 Hz probing whether the expected effects could be merely due to delivering alternating current, and a placebo stimulation (sham condition) where no current was delivered.

**Reviewer #2: This was a thorough revision and the authors have been very responsive to my feedback. They present interesting data from a sound and scientific approach that is a valuable contribution. In conclusion, I recommend its publication.**

**Reviewer #3: Benedikt Zoefel: I thank the authors for addressing all of my comments. I think they have produced a strong manuscript.**

**Answer:** We thank Reviewer 2 and Reviewer 3 for their positive responses and for having appreciated our efforts. We believe the previous round of comments we received from both Reviewers has considerably contributed in improving our manuscript.

**Additional remarks:**

We have realized an error was present in figure S4-B and we have now corrected it in the new version of the manuscript. Please note that statistical analyses concerning this measure (i.e. global phonological awareness) have not changed with respect to the previous version.
